# Exercise Early and Often: Effects of Physical Activity and Exercise on Women’s Bone Health

**DOI:** 10.3390/ijerph15050878

**Published:** 2018-04-28

**Authors:** Karen L. Troy, Megan E. Mancuso, Tiffiny A. Butler, Joshua E. Johnson

**Affiliations:** Department of Biomedical Engineering, Worcester Polytechnic Institute, Worcester, MA 01545 USA; memancuso@wpi.edu (M.E.M.); tbutler@wpi.edu (T.A.B.); jejohnson@wpi.edu (J.E.J.)

**Keywords:** bone mineral density, areal bone mineral density, volumetric bone mineral density, quantitative computed tomography, high resolution peripheral quantitative computed tomography, structure, mechanical loading, bone adaptation

## Abstract

In 2011 over 1.7 million people were hospitalized because of a fragility fracture, and direct costs associated with osteoporosis treatment exceeded 70 billion dollars in the United States. Failure to reach and maintain optimal peak bone mass during adulthood is a critical factor in determining fragility fracture risk later in life. Physical activity is a widely accessible, low cost, and highly modifiable contributor to bone health. Exercise is especially effective during adolescence, a time period when nearly 50% of peak adult bone mass is gained. Here, we review the evidence linking exercise and physical activity to bone health in women. Bone structure and quality will be discussed, especially in the context of clinical diagnosis of osteoporosis. We review the mechanisms governing bone metabolism in the context of physical activity and exercise. Questions such as, when during life is exercise most effective, and what specific types of exercises improve bone health, are addressed. Finally, we discuss some emerging areas of research on this topic, and summarize areas of need and opportunity.

## 1. Introduction

In 2011 over 1.7 million people were hospitalized because of a fragility fracture, and direct costs associated with osteoporosis treatment exceeded 70 billion dollars in the United States [1]. A woman just over age 50 in the United States has a 3.4%, 5.3%, and 6.8% risk of experiencing a fragility fracture within the next 10 years based on normal, low, and osteoporotic bone mass, respectively, evaluated by dual energy X-ray absorptiometry (DXA) T-scores [2]. Failure to reach and maintain optimal peak bone mass during adulthood is a critical factor in determining fragility fracture risk later in life. It has been estimated that an increase in peak bone mass of 10% would impart an additional 13 years of osteoporosis-free life for a typical older woman [3]. Although many effective pharmaceutical treatments have been developed to treat osteoporosis over the past three decades, prevention remains the best option.

Physical activity is a widely accessible, low cost, and highly modifiable contributor to bone health. Exercise transmits forces through the skeleton, generating mechanical signals, such as bone strain, that are detected by osteocytes. In healthy systems, signals related to strain magnitude and rate initiate a cascade of biochemical responses that locally and systemically increase bone turnover, resulting in net bone apposition. This is why the National Osteoporosis Foundation, International Osteoporosis Foundation, and other agencies recommend weight-bearing exercises for the prevention of osteoporosis [4,5,6,7].

Here, we review the evidence linking exercise and physical activity to bone health in women. Bone structure and quality are discussed, especially in the context of clinical diagnosis of osteoporosis. We review the mechanisms governing bone metabolism in the context of physical activity and exercise and summarize areas of need and opportunity. Questions such as, when during life is exercise most effective, and what specific types of exercises improve bone health, are addressed. Finally, we discuss some emerging areas of research on this topic.

## 2. Measurement of Bone Strength and Fracture Risk

A fracture occurs when the forces applied to a bone exceed its strength. Thus, bone strength is a critical factor that affects fracture risk. Bone tissue is a highly organized composite material comprised of type I collagen (23% dry weight) and ground substance (2% dry weight), covered with apatite mineral crystals (75% dry weight) [8]. Whole bone strength cannot be directly measured in a living person, but can be estimated indirectly. Strength depends on a number of factors, including the size, structure, and material properties of the bone tissue. Size and structural properties include cortical thickness, cross-sectional area, and moment of inertia. They also include microstructural variables that describe trabecular bone volume fraction, number, spacing, and heterogeneity, and cortical porosity. Bone material properties are often expressed as measures relating to volumetric bone mineral density (vBMD) in g/cm^3^, which has been related to mechanical stiffness [9,10,11]. However, the organization of the collagen and mineral components also play key roles in bone material behavior [12]. Collectively, all aspects of bone material and structure contribute to the mechanical strength of a given bone. And, all of these parameters change with age, resulting in age-related deterioration of bone strength [13].

Clinically, bone strength is usually assessed indirectly with dual energy X-ray absorptiometry (DXA). DXA uses X-rays to measure the total amount of mineral present in the imaged site—the bone mineral content (BMC), in grams. Because DXA produces a two-dimensional image similar to a plain radiograph, the projected area of the bone is measured in cm^2^. These two values are divided to calculate a ratio of BMC/area, or areal bone mineral density (aBMD, in g/cm^2^). aBMD is, in turn, expressed on a normalized scale in standard deviations as a T-score, relative to a young healthy sex- and race-matched population [14]. In this scale, a value of zero represents the average aBMD of a young, healthy adult, while negative values indicate below-average aBMD. The World Health Organization (WHO) defines osteoporosis as a T-score of −2.5 or lower (i.e., more than 2.5 standard deviations below the expected value for a young healthy adult).

DXA is limited, in that it provides only a two-dimensional measure, which is indirectly related to bone strength. Despite this shortcoming, aBMD explains 57% of the variance in hip fracture strength [15]. Combined with other epidemiologic factors such as family history, smoking status, and demographics, T-score is a significant predictor of future fracture [16,17]. As a result, many countries now recommend using fracture risk, calculated using the WHO’s country-specific Fracture Risk Assessment Tool (FRAX) calculator [2] as the basis for making treatment decisions for osteoporosis [4].

**Figure 1:**
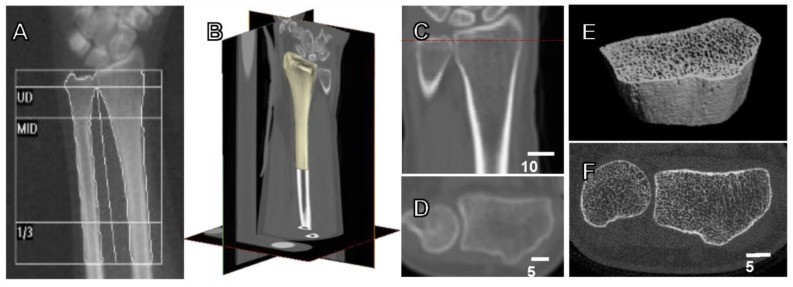
Current available methods for the assessment of bone strength and fracture risk. (**A**) Dual energy X-ray absorptiometry (DXA) forearm scan with standard ultradistal (UD), middle (MID) and one-third of arm length (1/3) regions, used to calculate aBMD (g/cm^2^). (**B**) 3D view of clinical computed tomography (CT) scan of the distal radius, with (**C**) coronal view containing dotted line indicating position of (**D**) transverse view. CT scan acquired at a transverse pixel size of 234 μm and slice thickness of 625 μm. (**E**) 3D view of high resolution peripheral quantitative CT (HRpQCT) image (**F**) of the distal radius, with isotropic voxel size of 82 μm.

Three-dimensional measures of bone, such as those derived from computed tomography (CT), provide a more complete picture of bone quality, but are less widely available clinically. A distinct advantage of quantitative CT analysis (QCT) is the ability to measure many of the parameters that directly contribute to fracture strength (Figure 1). As a result, QCT is a better predictor of fracture strength than DXA, explaining up to 66–79% of the variance in strength [15,18,19]. However, there are fewer large-scale, population-based studies reporting the relationship between QCT measures and fracture risk. In the past decade, high resolution peripheral quantitative CT (HR-pQCT), which has the ability to measure cortical and trabecular microstructure, has become increasingly common in research settings. Compared to DXA, the primary strength of QCT measures is the ability to determine the specific aspects of bone structure that change in response to treatment or disease.

## 3. Bone Adaptation and the Biological Basis for Why Physical Activity and Exercise Matters

Under most circumstances, bone adapts its structure to the typical mechanical environment to which it is exposed. Consistent with this phenomenon, a history of physical activity is associated with beneficial structural features in skeletally mature bone. Features such as greater cross-sectional area, bone mineral density (BMD), and moments of inertia collectively result in a stronger bone and have been observed in gymnasts versus nongymnasts [20,21], and between the dominant and nondominant arms of racquet sports players [22]. These observed differences are due to functional adaptation, the process where the cells within a bone modify its structure in response to loading.

Physical activity generates external (ground reaction and inertial) and internal (skeletal muscle) forces on the skeleton. These forces cause very small amounts of deformation in the bone tissue, resulting in mechanical strain (ε), a normalized measure of deformation. This mechanical strain, or a consequence of the strain such as fluid flow within the bone from one location to another, is sensed by osteocytes, the mechanosensitive cells that reside in bone. When unusual strains are sensed, osteocytes initiate an adaptive response through the action of osteoclasts, which resorb bone tissue and osteoblasts, then produces new bone tissue.

For a given external force, weak bones deform more, resulting in relatively large tissue strains, whereas strong bones experience low strains. This elicits a more robust biological, bone-building response in the weaker bone that eventually results in stronger bone—a phenomenon described by some as a “mechanostat” [23]—with bone having a mechanical set point similar to a thermostat. Although the actual process is understood to be far more complex than the analogy implies, the basic principle has been upheld through both retrospective and prospective observation. For example, bone adaptation in skeletally mature women has been observed to be site-specific and related to energy equivalent strain, with high strain regions experiencing more bone apposition than low strain regions [24].

Quantitative histomorphometry studies in humans and animal models have shown that in normal physiologic situations, bone is remodeled through the coordinated action of osteoclasts and osteoblasts. Remodeling takes place constantly, with 5% of adult cortical bone and 25% of trabecular bone turned over each year [8]. Osteoclasts are large, multinucleated cells responsible for bone resorption. They originate from mesenchymal stem cells and act within bone (cortical) and on bone surfaces (trabecular) to resorb tissue at a rate of 40 μm/day [8]. Osteoclast activation is controlled through the parathyroid hormone pathway [25], but the degree to which osteoclasts are able to target a specific location, versus acting at a random location, is not well known. There is evidence that local mechanical environment within the bone (e.g., bone strain, fluid shear flow, electromagnetic fields, the presence of microdamage, and other factors) influences osteoclast recruitment to a particular location [26].

Osteoblasts, which are responsible for laying down new bone tissue, generally follow osteoclasts to replace or modify the removed tissue. Beyond simply replacing bone tissue, osteoblasts can also add tissue to existing surfaces. It is important to note that osteoblasts add bone at a rate of about 1 μm/day [8]—substantially slower than bone is removed. Thus, even when the two cell types act in a coordinated fashion, too much osteoclast activation can result in net bone loss. Overactivation of osteoclasts has been implicated as a primary factor in post-menopausal bone loss, in part because estrogen inhibits osteoclast activation [27].

Although the relationship between mechanical signals and bone adaptation has been extensively studied in animals, the specifics are not well understood in humans due to difficulties in measuring both the stimulus and the change in bone structure noninvasively. Specific characteristics such as strain magnitude and rate [28,29], as well as underlying physiologic factors such as circulating hormones [30] and vitamin D concentration, collectively influence the bone adaptive response. A more detailed understanding of these factors would allow individuals who were likely to respond to biomechanical interventions for bone health to be identified, and would facilitate improved outcomes of such interventions.

## 4. When in Life Does Physical Activity and Exercise Matter the Most?

Physical activity is an essential component of a healthy lifestyle. While activity can be particularly beneficial for the gaining and maintenance of healthy strong bones in children and adolescence [31], a major determinant on how bones will respond to exercise depends primarily on age of the onset of the activity: prepubescent, early puberty, adolescence, young adult and mature. Variations in response to exercise have also been observed in sex, type of activity and duration of exercise, with bone response being somewhat site-specific.

**Figure 2:**
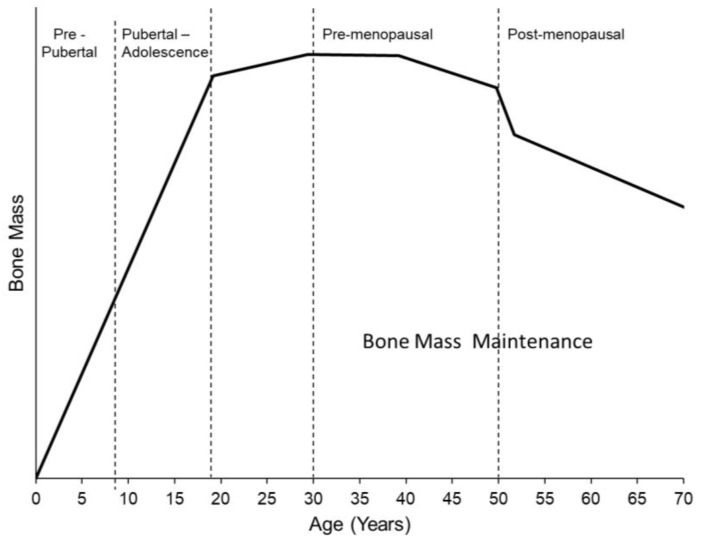
Typical pattern of age-related changes in bone mass, which is primarily accrued during the pre-pubertal and adolescent stages, reaches a lifetime peak at approximately 18 years of age, and declines sharply during perimenopause and steadily post-menopause.

In women, 80–90% of peak adult bone mass is accrued by age 16 [32], with nearly 50% of mass acquired during four circum-menarcheal years. Peak bone mass is obtained at approximately 18 years of age with growth maintained through the third decade [33,34] (Figure 2). Physical activity is a major factor in bone accrual and can significantly influence annual gains in bone density and mass during this period [35]. The bone of growing children is particularly sensitive to external factors like physical activity, which results in increased bone size and density that persist many years later.

High impact exercises, which generate large and rapid strains on the skeleton, appear to be most beneficial [36]. For example, six months of jumping exercises in adolescent girls and boys resulted in skeletal gains at the femoral neck and lumbar spine of from 1–6%, and 0.3% to 2%, respectively [37]. Bone strength improvements of 1–8% have been observed at loaded skeletal sites in children and adolescents aged 8–17 who did consistent weight bearing activities [38]. And, prepubescent children who exercised experienced greater changes in BMC and aBMD in the femoral neck and spine compared to those who did not exercise [35]. Physical activity in children increases bone mineral even when exercise duration is over a limited period of time [39,40,41,42,43]. 

Exercise and physical activity during growth lead to increases in bone size, density, and strength that persist for many years. Fuchs and Snow reported that after a bout of 7 months of high impact training and a 7-month follow-up period, an increase of 4% in femoral neck BMC and area was observed [41]. However, more importantly, these significant effects persisted even 8 years later [44]. Similar results were observed in 12.5 + 1.5 year old girls, in whom 9 months of high impact jumping, followed by 20 months of normal activity resulted in a 28% increase in BMC at the lumbar spine, which was 6% greater than a control group who did not jump [45]. During the accrual phase, adolescents and young adults have the capacity to gain bone mass, which needs to last throughout the lifetime. Although exercise during this period enhances normal gains in bone mass and geometry that occur during growth [46,47], these improvements may not last into maturity, due to effects of remodeling [48,49].

Bone mass generally peaks around the 3rd decade of life [33], with external factors such as exercise playing a role in the incremental increases in mass and geometry occurring through the life span. In older adults (≥ 60 years), bone mass cannot be gained through physical activity, but bone loss can be prevented. After menopause, women typically experience annual losses in bone mass and strength of −0.5%/year and −2.5%/year, respectively [50]. However, sustained physical activity has a beneficial effects on bone and works to attenuate bone loss [51]. Reviews and meta analyses specifically looking at aBMD at the proximal femur and/or the lumbar spine in the aging population suggest that a combination or single use of resistance training and weight bearing impact exercise prevents bone loss after menopause [52,53,54,55,56,57]. Bone strength increases of 0.5% to 2.5% have also been observed in premenapausal women who participate in sustained weight bearing resistance exercises. High-impact loading exercise also benefits bone mass and geometry in this population [58]. When early postmenopausal women exercised for 12 months or longer, they experienced small increases in trabecular and cortical bone volumetric BMD in the tibial shaft [59].

## 5. Which Specific Types of Physical Activity Are Best for Bone?

The National Osteoporosis Foundation, International Osteoporosis Foundation (NIAMS), and other agencies recommend weight-bearing exercises for the prevention of osteoporosis [5,6,7]. These include high impact exercises such as jumping, aerobics, and running, as well as lower impact exercises such as walking and weight training. The evidence for high impact exercises is the most robust, although weight training also appears to be effective in pre-menopausal women. For example, repeated impact and resistive loading, i.e., plyometric training (bounding up and down, or jumping/hopping) [41] and weight lifting, have been shown to have positive effects on bone at every age range [60]. A recent small clinical trial piloting high intensity resistance and impact training demonstrated significant improvements in proximal femur and lumbar spine density and geometry in postmenopausal women, warranting further investigation [61]. During adolescence, resistive exercise can increase bone strength [31]. In middle age and post puberty, resistive training is effective at attenuating loss of bone mass and density [60]. A varied exercise regimen that includes a mix of high impact and weight-bearing training, and aerobic training, may prevent senile bone loss [51] and may increase hip and spine BMD [62]. In the aging population, walking has only marginal or nonexistent effects on bone [51]. Lower impact activities such as cycling, yoga, and swimming, which are typically recommended as lifetime fitness activities for aging populations, are generally not considered osteogenic. For example, competitive female cyclists experienced −1.4% and −1.1% changes in BMD at the hip and lumbar spine during a 12 months study period [63]. Swimming is generally associated with similar or slightly lower BMC and BMD in the lower limbs [64,65]. A small recent study in postmenopausal women showed that regular practice of certain yoga postures may modestly improve monthly change in spine, but not femur BMD [66]. These exercises could potentially be combined with resistive on-land weight bearing activity to better target bone health. However, if these activities are not coupled with weight-bearing activities, they will not provide the magnitude of loading necessary to maintain bone mass and density [67].

Although vigorous, high-impact exercise is best for increasing BMD, there are other considerations to be made when selecting an appropriate exercise program. In general, mechanical loading during exercise does not negatively affect joint health, and is in fact recommended for the improvement of osteoarthritis (OA) symptoms [68]. However, in OA patients who are obese or have abnormal joint biomechanics due to history of traumatic injury or surgery, high-impact loading can exacerbate joint degradation [68]. Additionally, it has been shown that mechanical loading has a different effect on the structure of healthy versus diseased cartilage. In healthy cartilage, increased joint moments are associated with increased thickness and health, whereas in individuals with established OA, increased joint moments are associated with decreased cartilage thickness [69]. Running, in particular, can improve cardiovascular health, muscle strength, and bone health, but is commonly implicated as being high risk for joint injury. However, a recent meta-analysis that included 17 studies and over 100,000 individuals found that only 3.5% of recreational runners had OA, vs. 10.2% of sedentary individuals and 13.3% of competitive runners [70]. Therefore, while regular and recreational-level high-impact exercise throughout one’s lifetime does not increase the risk of OA, initiating a high-impact exercise intervention after joint degradation is present may negatively impact disease progression. Additionally, individuals with cardiovascular health problems may not be able to engage in vigorous exercises such as those recommended here for improving BMD. Nevertheless, resistive and low-impact, weight bearing exercises have been shown to improve balance and reduce fall incidence in older adults with low BMD, thereby decreasing fracture risk without necessarily increasing BMD directly [71].

## 6. Emerging Areas of Research about Exercise and Women’s Bone Health

### 6.1. Measuring Bone Loading In Vivo

There remains a disconnect between animal studies, which consider bone tissue strains during loading, and human trials, which typically only measure forces applied external to the body. Direct measurement of bone strain requires highly invasive methods. A small number of studies, the first published in 1975 [72], have used strain gauges applied to the outer bone surface to measure normal and shear strain during various activities [73,74,75]. This technique is limited to a small region of the outer surface of sites with minimal soft tissue, and strain gauges cannot be left in the body long-term. More recently, Yang et al. [76,77] developed a method for measuring tibia deformations by calculating displacement of small optical markers on bone screws inserted into the periosteal bone surface. While they have produced valuable data that can be used to validate less invasive estimates of bone strain, these techniques are not feasible to implement in the clinical setting.

**Figure 3:**
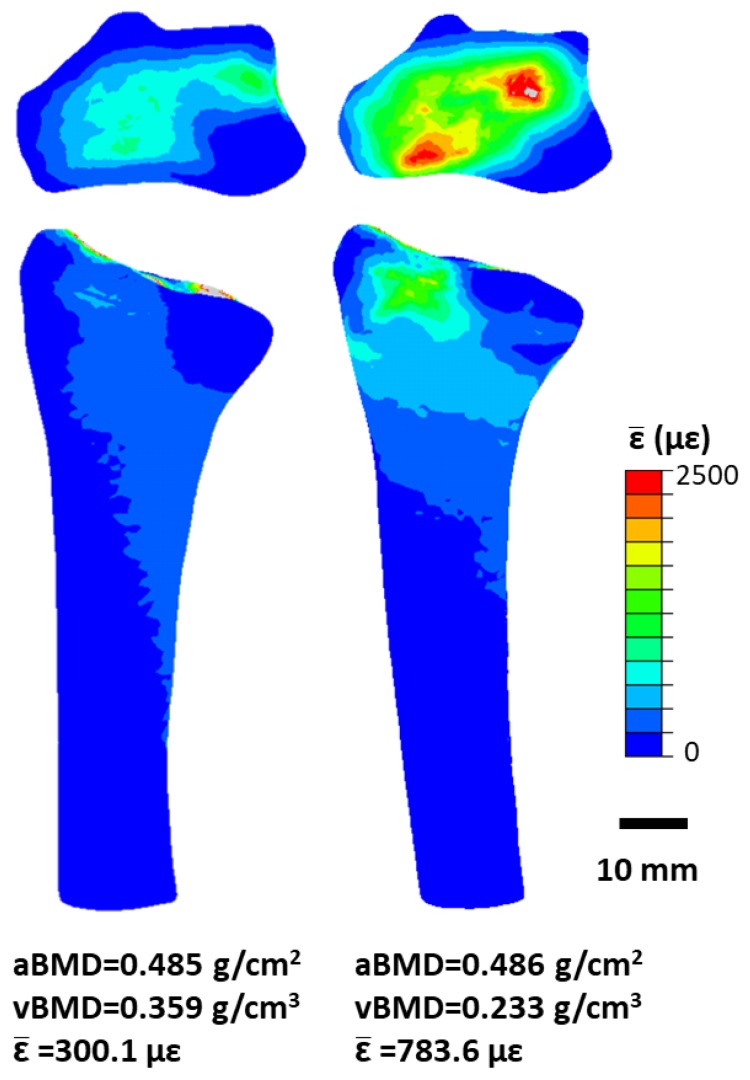
Bone strain (expressed as energy-equivalent strain, $\bar{\varepsilon }$ [24]) in the distal radius and transverse slice with maximum cross-sectional area. Percent difference in aBMD is 0.21%, while percent difference in vBMD and mean energy equivalent strain in the 9.375 mm ultradistal region is are 42.68% and 89.23%, respectively.

Our work has used a combination of force sensors and validated, patient-specific, finite element (FE) models [78]. The finite element method is a numerical modeling technique that can be used to understand how complex structures behave under various types of mechanical loads. We use FE models to estimate physiological bone strain during an upper-extremity loading intervention wherein volunteers lean onto the palm of their hand to reach a target force [79]. The compressive force applied during this simple task is measured using a uniaxial load cell and simulated using a CT-based FE model of the radius, scaphoid and lunate. We have shown that among premenopausal women with normal bone mineral density (T-score [−2.5,1.0]), bone strain, which stimulates bone adaptation, is highly variable even when the same external force is applied to the hand (Figure 3) [80]. We believe that in the future, exercise interventions would be more successful if individual differences in anatomy were considered, to generate specific bone strains. This is based on our data in young premenopausal women, which shows that increases in BMC occur preferentially in local regions of high strain [24]. These results underline the importance of further developing techniques to estimate subject-specific bone strain to understand the mechanism of functional bone adaptation in humans.

### 6.2. 3D and High Resolution Imaging of Bone

Although osteoporosis is clinically defined using DXA, there is substantial ongoing research focused on imaging bone in three dimensions and at increasingly smaller scales. QCT is used to calculate vBMD, BMC and bone volume from clinical CT scans. Typically, this technique can detect structural features around 0.5 to 2 mm or smaller. Additionally, 3D bone surfaces can be generated from segmented QCT images and converted to finite element models to estimate bone strength [81]. QCT-based FE outcomes are superior predictors of fracture strength compared to DXA at the tibia [18] and femur [19]. Additionally, QCT-based FE analysis has been approved by the United States Food and Drug Administration to estimate and monitor fracture risk during osteoporosis treatment. Thus FE as an alternative outcome for clinical trials [82,83], rather than fractures, may reduce the costs and time associated with bringing new osteoporosis drugs to market. The primary concern in adopting QCT in the clinic is whether the added value in fracture risk prediction outweighs the increased radiation dose and cost required to obtain large 3D scans. However, phantomless calibration techniques have been introduced recently to enable the retrospective analysis of existing CT scans [84].

**Figure 4:**
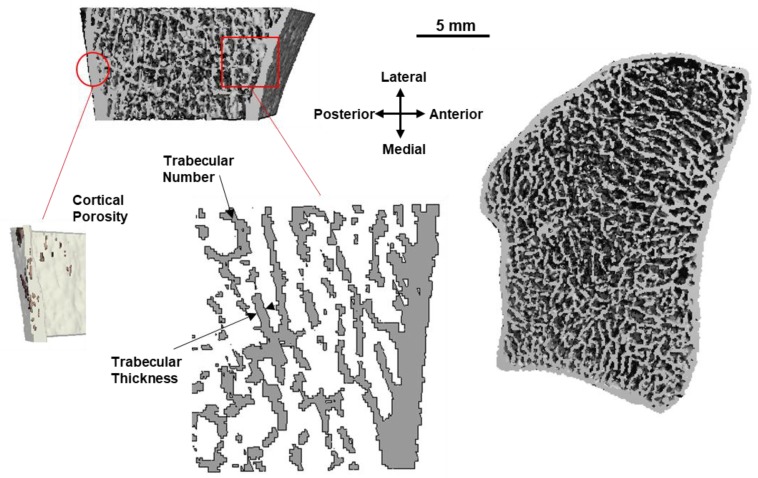
Distal radius microstructure acquired using HR-pQCT viewed from the transverse plane (right) and sagittal cross-section (top left). Insets show example measurements of compartment-specific cortical (porosity) and trabecular (number, thickness) microstructure parameters, made possible through this emerging technology.

HR-pQCT has enabled the in vivo imaging of human bone microstructure [85]. First- and second-generation [86] HRp-QCT scanners have 82 and 61 μm voxel sizes, respectively, allowing for the detection and measurement of individual trabeculae. Currently, HR-pQCT is limited to small regions in the distal tibia and radius, with second-generation scanners allowing for scanning of the knee [87]. HR-pQCT has contributed to the understanding of how age-related bone loss occurs, showing that post-menopausal women tend to experience loss of trabeculae but increased trabecular thickness in the radius [88] and trabecularization of the endosteal surface and increased cortical porosity in the radius and tibia (Figure 4) [88,89]. Additionally, FE models based on HR-pQCT scans have been used to estimate failure load of the 9-mm scanned region under platen compression, simulating a mechanical test of the bone [90,91].

One long-term goal is to be able to “design” an exercise for bone health, to produce an osteogenic response. To accomplish this, the strains that produce an osteogenic response must be known, and the mechanical strains that occur in a bone during a candidate exercise must be quantified. FE models are useful for estimating strains within the bone of a living person. However, we have shown that models that simulate platen compression, which often used to estimate bone strength, do not accurately replicate the strains that occur during physiologic loading [92]. If FE models based on these images are to be useful for predicting bone strain during an exercise, it is important to include accurate (physiological) boundary conditions [93]. Additional research is aimed at predicting bone fracture behavior by including material and geometric nonlinearity [94] and fracture mechanics [95,96] within the models. Ultimately, a combination of imaging techniques at multiple scales is likely required to obtain the most complete understanding of a patient’s susceptibility to fracture.

### 6.3. Detecting the Short-Term Response to Osteoporosis Treatment

Measuring a patient’s short-term biological response to loading may enable the personalized optimization of exercise interventions. Several serum and urine bone turnover markers have been used to assess the effect of exercise on bone metabolism. Bone formation markers indicative of osteoblast activity include bone-specific alkaline phosphatase (BALP), osteocalcin (OC), and procollagen type I N propeptide (PINP) and procollagen type I C propeptide (PICP). Bone resorption markers indicative of osteoclast activity include C-terminal and N-terminal cross-linked telopeptides of type I collagen (CTX and NTX), tartrate-resistant acid phosphatase 5b (TRAP5b), deoxypyridinoline, and pyridinoline. The International Osteoporosis Foundation (IOF) and International Federation of Clinical Chemistry and Laboratory Medicine (IFCC) [97], as well as the National Bone Health Alliance (NBAA) [98] suggest PINP and CTX measured from blood serum be used as reference markers of formation and resorption, respectively. These groups also highlight the need for standardization of sample collection and laboratory assays and for reference ranges for each marker before bone biomarkers can be used widely to make treatment decisions.

Bone turnover markers have been used in several studies to assess the short-term effect of exercise on bone metabolism. Studies have focused on prepubescent girls [99,100], pre-menopausal [101,102,103,104,105], and post-menopausal women [106,107,108,109], and have looked at the short- and long-term biomarker response to exercise. Of particular interest to monitoring the biological response to mechanical loading is sclerostin, the protein product of the *SOST* gene in osteocytes. Sclerostin is an antagonist to Wnt signaling, decreasing bone formation by osteoblasts and increasing osteoclast activity via osteoprotogerin regulation. Animal models have shown that regulation of local sclerostin expression is sensitive to mechanical loading [110], and that local bone strains correlate to decreased sclerostin expression and increased bone formation [111]. Therefore, sclerostin may be a valuable biomarker in the assessment of existing and novel exercise interventions.

### 6.4. Interactions between Drugs for Osteoporosis and Exercise

Several studies have aimed to determine whether combined pharmaceutical/loading therapies are more effective than either treatment alone. This idea stems from the belief that an optimal osteoporosis treatment should both decrease resorption by osteoclasts and increase formation by osteoblasts. The majority of currently prescribed pharmaceuticals, with the exception of teriparatide, slow bone loss but are not anabolic. As mechanical loading has been shown to promote bone-turnover-favoring formation, it is thought that a combination of antiresorptives and exercise loading may have an additive effect on bone health. A meta-analysis of seven randomized controlled trials compared antiresorptive or hormone therapy alone (*n* = 215), with exercise plus antiresorptive or hormone therapy (*n* = 205). The authors found that patients who combined exercise with antiresorptive (alendronate or risedronate) or hormone therapy (conjugated estrogen alone, or estrogen plus medroxyprogesterone acetate) had significantly greater increases in lumbar spine bone mineral density compared to those who did not also exercise (standard mean difference 0.55) [112]. In support of this finding, another meta-analysis of nine studies (total *n* = 1248) comparing exercise alone versus exercise plus some form of antiresorptive or hormone therapy, found that the combination therapy resulted in significantly greater increases to lumbar spine aBMD. However, differences were insignificant in the proximal femur, suggesting that the interaction between loading and pharmaceuticals may be site-specific and depend on loading modality [53]. A definitive conclusion on the combined effects of pharmaceuticals and exercise loading requires better methods to measure and monitor loading, and this effect may vary with sex and age.

## 7. Conclusions

Physical activity is an important contributor to bone quality. Based on evidence from controlled clinical trials and meta-analyses (randomized/nonrandomized), the following recommendations can be made for physical activity and exercise.

1. Adolescent and prepubertal girls can derive the greatest benefit from bone-loading exercise. In this age group, exercise is an effective means of increasing peak bone mass, which provides lifelong fracture protection.
2. High-impact exercises such as jumping or hopping, or resistance training combined with high- or odd-impact activities, are most consistently effective for bone.
3. Two to four short (30 min/day or less) exercise sessions per week over a prolonged period are required to maintain or improve bone.
4. For older women who have risk factors that prevent them from participating in high-impact activities, other weight-bearing activities such as resistance training, specific yoga postures, or walking, may maintain or improve bone.
5. Other activities that preserve or improve mobility and strength are also beneficial, because they reduce fall risk, thereby reducing fracture risk.

There is a general consensus that high-impact (high-intensity) loading is beneficial for bones. The benefits of jumping, an impact loading activity, are also more evident at the hip than the spine [113]. Impact activities such as unilateral hopping that produce similar ground reaction forces as jumping have a positive effect over a prolonged period (at least 6 months) [114]. High-impact loading combined with other exercises that produce large joint reaction forces (such as resistance training) have a positive effect on bone [52,53,57,115,116,117]. Also, a combination of high- and odd-impact loading appears to be favorable [52,115,116] as opposed to high-impact or odd-impact or resistance training alone [53,115].

Bone response to mechanical loading is greatest in growing and adolescent children [42]. In menopausal women, the effect of combined exercise interventions appears dependent on skeletal site and age [62,118]. The recommended intensity of impact loading activities varies depending on the level of risk for fragility fracture (low-risk: > 4 BW; moderate-risk: > 2 BW; high-risk: 2–3 BW as tolerated [119]. The frequency of exercise needed to observe a positive effect is not trivial, particularly when considering the elderly population. Based on a long-term trial, the minimum effective frequency was determined to be two sessions/week over a 16-year period [120], and is even higher for impact activities alone (minimum four sessions/week) [119]. Brief (less than 30 min) high-impact activities have a positive effect mainly on femoral neck BMD, but not on lumbar spine BMD [121]. The effect of walking (low-impact) is only inconsistently positive at the femoral neck, provided that the intervention exceeds 6 months [54,122]. However, epidemiological data that suggest small hip BMD gains even with decreases in BMI in those increasing exercise to 30 min walking a day [118]. Additionally, walking independently contributes to fracture prevention by helping with fall avoidance [123].

There is potential for bias in meta-analyses and there exists a range of methodological and reporting inconsistencies (heterogeneity) between trials. Therefore, existing data should be interpreted with caution. While the effects of physical activity on BMD may be modest [124], they have clinically significant implications in terms of reduction in long-term fracture risk. For example, high-impact progressive resistance training was associated with a relative increase of 1% in lumbar spine BMD [57]. However, these small changes are estimated to reduce the 20-year osteoporotic fracture risk at any site by 10% [124]. Overall, physical activity appears to have a positive effect on bone health [125,126]. However, further work is needed to elucidate the specific factors that influence bone parameters for physical activity and exercise to contribute as a successful patient-specific intervention tool.
